# Supplementary material for: vardictcpp: a C++ reimplementation of VarDict variant caller
Source: GigaByte. 2026 Oct 20;2026:gigabyte194. doi: 10.46471/gigabyte.194 (PMC13720296; doi:10.46471/gigabyte.194)
Supplement: Peer review history

# Reviewer report: Review MS


Reviewer name and names of any other individual's who aided in reviewerRuining DongDo you understand and agree to our policy of having open and named reviews, and having your review included with the published manuscript. (If no, please inform the editor that you cannot review this manuscript.)YesIs the language of sufficient quality?YesPlease add additional comments on language quality to clarify if neededIs there a clear statement of need explaining what problems the software is designed to solve and who the target audience is? YesAdditional CommentsIs the source code available, and has an appropriate Open Source Initiative license <a href="https://opensource.org/licenses" target="_blank">(https://opensource.org/licenses)</a> been assigned to the code?YesAdditional CommentsAs Open Source Software are there guidelines on how to contribute, report issues or seek support on the code?YesAdditional CommentsIs the code executable?YesAdditional Commentstesting env: AppleClang 21 (C++17) on Apple Silicon (arm64), cmake v4.4.2, htslib v1.24.Is installation/deployment sufficiently outlined in the paper and documentation, and does it proceed as outlined?NoAdditional CommentsThe deployment instructions are outlined in the GitHub readme. The paper has no information of installation/deployment.Is the documentation provided clear and user friendly?YesAdditional CommentsThe paper does not include a detailed documentation, but it provides a github link to the Vardictcpp under section Availability of source code and requirements. The github repository contains a well-structured documentation including quick start code blocks and clear comparison tables.Is there enough clear information in the documentation to install, run and test this tool, including information on where to seek help if required?NoAdditional CommentsIn the github documentation, there are instructions to install via conda which can be updated with different installation env. For running, the doc lists three use cases but lacks a detailed table defining what each flag does. There is no dedicated help seeking information in the documentation. It would be ideal to specify the best way to seek help, such as the github issue page.Is there a clearly-stated list of dependencies, and is the core functionality of the software documented to a satisfactory level?YesAdditional CommentsHave any claims of performance been sufficiently tested and compared to other commonly-used packages? YesAdditional CommentsI have tested Vardict using 1, 2, 4, and 8 threads locally on Apple silicon using the test data, and the performances matched the claims.Is test data available, either included with the submission or openly available via cited third party sources (e.g. accession numbers, data DOIs)?YesAdditional CommentsAre there (ideally real world) examples demonstrating use of the software? YesAdditional CommentsIs automated testing used or are there manual steps described so that the functionality of the software can be verified?YesAdditional CommentsThere is a CI pipeline set up in the github repository automatically run tests across ubantu and macos with gcc and clang. However, as of 20 August 2026, the ci status is failing. The error is: macos-13 / x86_64 / clang. The job has exceeded the maximum execution time while awaiting a runner for 24h0m0s.Any Additional Overall Comments to the AuthorThere’s a discrepancy in byte-identical rates between GitHub page documentation and the paper. For Validation vs VarDictJava 1.8.3, the GitHub page reports a full-row byte-identical rate of 98.3% using a small synthetic sample while the paper claims 100% across five real WES samples. The GitHub page attributes the 1.7% difference to multi-variant and MNP-adjacent edge cases. This information is important and should be discussed in the paper. Real-world applications involve such variants and users need to be aware of the potential for different outcomes.RecommendationMinor Revisions


# Reviewer report: Review MS


Reviewer name and names of any other individual's who aided in reviewerQingxiang GuoDo you understand and agree to our policy of having open and named reviews, and having your review included with the published manuscript. (If no, please inform the editor that you cannot review this manuscript.)YesIs the language of sufficient quality?YesPlease add additional comments on language quality to clarify if neededIs there a clear statement of need explaining what problems the software is designed to solve and who the target audience is? YesAdditional CommentsIs the source code available, and has an appropriate Open Source Initiative license <a href="https://opensource.org/licenses" target="_blank">(https://opensource.org/licenses)</a> been assigned to the code?YesAdditional CommentsAs Open Source Software are there guidelines on how to contribute, report issues or seek support on the code?YesAdditional CommentsIs the code executable?Unable to testAdditional CommentsIs installation/deployment sufficiently outlined in the paper and documentation, and does it proceed as outlined?Unable to testAdditional CommentsIs the documentation provided clear and user friendly?YesAdditional CommentsIs there enough clear information in the documentation to install, run and test this tool, including information on where to seek help if required?YesAdditional CommentsIs there a clearly-stated list of dependencies, and is the core functionality of the software documented to a satisfactory level?YesAdditional CommentsHave any claims of performance been sufficiently tested and compared to other commonly-used packages? YesAdditional CommentsThe performance claims are supported by comparisons with VarDictJava and several commonly used variant callers. However, the benchmark settings and commands should be described more clearly to ensure reproducibility, and the runtime discrepancy between the text and Table 3 should be corrected.Is test data available, either included with the submission or openly available via cited third party sources (e.g. accession numbers, data DOIs)?YesAdditional CommentsAre there (ideally real world) examples demonstrating use of the software? YesAdditional CommentsIs automated testing used or are there manual steps described so that the functionality of the software can be verified?YesAdditional CommentsAny Additional Overall Comments to the AuthorThis Technical Release presents vardictcpp, a C++ reimplementation of VarDictJava that aims to keep the same variant calling behavior while using less time and memory. The performance gains are promising, and the tool could be useful for users who run VarDict routinely or need to process many samples with limited computing resources. I think the manuscript is generally straightforward, but a few points still need clarification: especially how thoroughly the C++ implementation was validated against VarDictJava, how the multi-threaded mode works, and whether all benchmark comparisons can be reproduced from the information provided. There are also a few small inconsistencies in the text and tables that should be corrected. 1. “While the boom in artificial intelligence has driven the increase in memory prices, agentic coding assistants have proven useful for re-implementing software quickly…” - This paragraph reads more like Implementation + Results. I suggest moving the main technical details to the Implementation section and keeping at most one sentence here in the Statement of Need. 2. “Driven by demand from artificial intelligence, memory prices have reportedly risen sharply, reaching 448% of the previous year’s level in Germany in the summer of 2026 [3].” - Reference 3 states: “Wccftech, German DDR5 Memory Prices Explode to 448% of July 2025 Levels; 2025.” There appears to be at least a discrepancy in the year, which the authors should double-check. 3. “Each core module of VarDictJava ... was translated individually and tested against reference outputs generated by running VarDictJava on shared synthetic and real-world BAM datasets.” - The authors should provide more details on which variant types, parameters, and edge cases were covered by these validation tests, and indicate where the corresponding test data or scripts are available. Since “functional equivalence” is a central claim of this study, stating only that the implementation was “tested against reference outputs” is not sufficient to assess the coverage of the validation. 4. “Because vardictcpp reimplements VarDict’s algorithm exactly...” - I think “reimplements VarDict’s algorithm exactly” is too strong. 5. Implementation The 8-thread performance is presented as one of the main results, but this section does not explain how parallelization is implemented in vardictcpp. Please briefly describe how tasks are distributed across threads, how the thread number is specified, and whether multi-threaded execution affects output order or result consistency. 6. “vardictcpp completed slightly faster at 10 seconds but required 10 GB of memory.” - Table 3 reports a runtime of 2:03–2:01 for vardictcpp in the whole-chromosome experiment, whereas the text reports 10 seconds. 7. “six variant callers have been tested on the HG002 sample of genome in a bottle (GIAB) consortium” - Figure 2 actually indicates HG002 chr20 rather than the entire HG002 genome. I suggest clarifying this in the text and providing the truth set, benchmark regions, and method used to calculate the F1 score. Otherwise, this part of the analysis is difficult to reproduce. Minor issue: 8. Page 2, Statement of Need - “VarDictJava[4, 5] uses the −R parameter…”, There is no space before the citation. 9. “Considering the multi-core with 8 cores, vardictcpp is computed in 19 seconds…” - Should be “With 8 cores, vardictcpp completes the analysis in 19 seconds...” 10. “German DDR5 Memory Prices Explode To 448% Of July 2025 Levels As Every Kit Gets Swept Into RAMpocalypse” This report seems to have been published in 2026 instead of 2025. Please double-check.RecommendationMinor Revisions


# Editor report: Accept


Editor’s AssessmentThe manuscript is ready for formal acceptance.Editor’s AssessmentThe manuscript is ready for formal acceptance.
